# Chemotherapy-Related Toxic Effects and Quality of Life and Physical Functioning in Older Patients

**DOI:** 10.1001/jamanetworkopen.2023.39116

**Published:** 2023-10-23

**Authors:** Joosje C. Baltussen, Nienke A. de Glas, Yara van Holstein, Marjan van der Elst, Stella Trompet, Anna Uit den Boogaard, Willeke van der Plas-Krijgsman, Geert Labots, Cynthia Holterhues, Jessica M. van der Bol, Lemonitsa H. Mammatas, Gerrit-Jan Liefers, Marije Slingerland, Frederiek van den Bos, Simon P. Mooijaart, Johanneke E. A. Portielje

**Affiliations:** 1 Department of Medical Oncology, Leiden University Medical Center, Leiden, the Netherlands; 2 Department of Gerontology and Geriatrics, Leiden University Medical Center, Leiden, the Netherlands; 3 Department of Internal Medicine, Haga Hospital, The Hague, the Netherlands; 4 Department of Geriatrics, Reinier de Graaf Hospital, Delft, the Netherlands; 5 Department of Medical Oncology, Reinier de Graaf Hospital, Delft, the Netherlands; 6 Department of Surgery, Leiden University Medical Center, Leiden, the Netherlands

## Abstract

**Importance:**

Although older patients are at increased risk of developing grade 3 or higher chemotherapy-related toxic effects, no studies, to our knowledge, have focused on the association between toxic effects and quality of life (QOL) and physical functioning.

**Objective:**

To investigate the association between grade 3 or higher chemotherapy-related toxic effects and QOL and physical functioning over time in older patients.

**Design, Setting, and Participants:**

In this prospective, multicenter cohort study, patients aged 70 years or older who were scheduled to receive chemotherapy with curative or palliative intent and a geriatric assessment were included. Patients were treated with chemotherapy between December 2015 and December 2021. Quality of life and physical functioning were analyzed at baseline and after 6 months and 12 months.

**Exposures:**

Common Terminology Criteria for Adverse Events grade 3 or higher chemotherapy-related toxic effects.

**Main Outcomes and Measures:**

The main outcome was a composite end point, defined as a decline in QOL and/or physical functioning or mortality at 6 months and 12 months after chemotherapy initiation. Associations between toxic effects and the composite end point were analyzed with multivariable logistic regression models.

**Results:**

Of the 276 patients, the median age was 74 years (IQR, 72-77 years), 177 (64%) were male, 196 (71%) received chemotherapy with curative intent, and 157 (57%) had gastrointestinal cancers. Among the total patients, 145 (53%) had deficits in 2 or more of the 4 domains of the geriatric assessment and were classified as frail. Grade 3 or higher toxic effects were observed in 94 patients (65%) with frailty and 66 (50%) of those without frailty (*P* = .01). Decline in QOL and/or physical functioning or death was observed in 76% of patients with frailty and in 64% to 68% of those without frailty. Among patients with frailty, grade 3 or higher toxic effects were associated with the composite end point at 6 months (odds ratio [OR], 2.62; 95% CI, 1.14-6.05) but not at 12 months (OR, 1.09; 95% CI, 0.45-2.64) and were associated with mortality at 12 months (OR, 3.54; 95% CI, 1.50-8.33). Toxic effects were not associated with the composite end point in patients without frailty (6 months: OR, 0.76; 95% CI, 0.36-1.64; 12 months: OR, 1.06; 95% CI, 0.46-2.43).

**Conclusions and Relevance:**

In this prospective cohort study of 276 patients aged 70 or older who were treated with chemotherapy, patients with frailty had more grade 3 or higher toxic effects than those without frailty, and the occurrence of toxic effects was associated with a decline in QOL and/or physical functioning or mortality after 1 year. Toxic effects were not associated with poor outcomes in patients without frailty. Pretreatment frailty screening and individualized treatment adaptions could prevent a treatment-related decline of remaining health.

## Key Points

**Question:**

What is the association between grade 3 or higher chemotherapy-related toxic effects and quality of life (QOL) and/or physical functioning at 6 months and 12 months in patients aged 70 years or older treated with chemotherapy?

**Findings:**

In this multicenter cohort study that included 276 patients aged 70 years or older treated with chemotherapy, grade 3 or higher toxic effects were associated with a composite end point (decline in QOL and/or physical functioning or mortality) in patients with frailty. Grade 3 or higher toxic effects were not associated with the composite end point in patients without frailty.

**Meaning:**

The findings suggest that, among patients with frailty, the occurrence of grade 3 or higher toxic effects was associated with a decline in QOL and/or physical functioning or mortality after 1 year, highlighting the importance of pretreatment frailty screening and individualized treatment adaptions.

## Teaser

This cohort study assesses the association between grade 3 or higher chemotherapy-related toxic effects and quality of life (QOL) and/or physical functioning or mortality at 6 months and 12 months among patients 70 years or older receiving chemotherapy.

## Introduction

With the aging of the global population, the number of older patients with cancer will continue to rise in the coming years.^1^ Despite this demographic shift, current oncologic practices are still based on trials in which older patients are underrepresented,^2^ and relevant end points for older patients, such as quality of life (QOL) and functional independence,^3,4^ have often not been measured. As a result, data to guide anticancer treatment in older patients remain limited.

In daily practice, clinicians and patients weigh the expected benefit of chemotherapy against the risk of toxic effects. This is particularly true for older patients, who have an increased risk of developing toxic effects due to decreased bone marrow reserve, reduced renal and liver function, and multimorbidity,^5,6,7^ resulting in unplanned hospitalization and early treatment discontinuation. Predominantly older patients who are frail are at risk of these outcomes,^8^ since frailty, caused by a cumulative decline across multiple organ systems, may result in a decreased ability to tolerate chemotherapy.^9^

Chemotherapy-related toxic effects potentially may have a negative impact on QOL and physical functioning and may even threaten independence. Although studies have investigated the association of toxic effects with long-term QOL and physical functioning,^10,11,12,13^ none of these studies, to our knowledge, have specifically focused on the older population. Therefore, we conducted a cohort study to investigate the association between grade 3 or higher chemotherapy-related toxic effects and QOL and physical functioning at 6 months and 12 months in older patients with cancer.

## Methods

Patients in this study were included in the prospective, ongoing Triage of Elderly Needing Treatment (TENT) cohort. The study was approved by the medical ethics committee of the Leiden University Medical Center and registered at the Dutch Trial Register (NL8107). Written informed consent was obtained from all patients. Extensive details of the TENT study were previously published.^14,15^ This study followed the Strengthening the Reporting of Observational Studies in Epidemiology (STROBE) reporting guideline for cohort studies.^16^

For this analysis, all patients aged 70 years or older with any malignant neoplasm who were scheduled to receive chemotherapy in 3 hospitals between December 2015 and December 2021 were selected. Patients who received chemotherapy with either curative or palliative intent within 90 days after inclusion and a baseline geriatric assessment (GA) were included. Patients who had already received 1 or more chemotherapy cycles before inclusion or were eventually treated in another hospital were not eligible. Concomitant radiotherapy, targeted therapy, immunotherapy, or surgery was allowed.

Before treatment initiation, participants underwent a geriatric frailty screening, consisting of the Geriatric 8 questionnaire^17^ and the Six-Item Cognitive Impairment Test.^18^ If one of the screening tools showed risk of frailty,^14^ participants underwent a comprehensive GA (CGA). If neither of the screening tools was positive, a GA was performed by telephone.

Functional status was assessed by the Katz Index of Independence in Activities of Daily Living (ADL)^19^ (scores range from 0 to 6, with higher scores indicating patient independence, with scores ≤2 indicating abnormal patient dependence). The Lawton Instrumental ADL (IADL)^20^ was used to assess independence (scores range from 0 to 5 for men and from 0 to 8 for women, with higher scores indicating high function and independence; the cutoff for abnormal dependence was ≤4 for men and ≤7 for women). Quality of life was assessed with the EuroQoL 5-Dimension 3-Level (EQ-5D-3L) questionnaire and visual analogue scale (EQ-VAS).^21^ We converted EQ-5D-3L scores into an index score,^22^ with a maximum score of 1 indicating the best health state. The Mini Nutritional Assessment^23^ was used to screen nutritional status (scores range from 0 to 14, with higher scores indicating normal nutritional status; the cutoff for risk of malnutrition was <12 points). The Patient Health Questionnaire-2^24^ was used to screen for anxiety and depression (scores range from 0 to 6, with higher scores indicating likelihood of depressive disorder; the cutoff of ≥3 points indicates major depressive disorder is likely).^24^ Comorbidities were scored with the Charlson Comorbidity Index (CCI).^25^ Six and 12 months after treatment initiation, participants were contacted by telephone to obtain the ADL, IADL, EQ-5D-3L, and EQ-VAS questionnaires. Patients were followed up for 1 year or until death. Data on mortality were gathered from digital patient files or municipal registries.

Similar to a previous publication,^15^ we defined frailty according to the somatic, functional, psychological, and social domains. If 1 or more tests in a domain were scored as abnormal, the domain was considered abnormal. Participants who scored abnormally on 2 or more of 4 domains were classified as frail. Comorbidity (CCI ≥1), polypharmacy (≥5 medicines), and malnutrition (Mini Nutritional Assessment) made up the somatic domain. The functional domain included falls within the past 6 months, institutionalization, and functional dependency (ADL and IADL). The psychological domain included dementia, history of delirium, and cognitive impairments (Six-Item Cognitive Impairment Test). The social domain was considered abnormal when patients lived alone. To validate this definition, we also measured frailty with the 10-Item Frailty Index Based on a Comprehensive GA (FI-CGA-10),^26^ which has been validated in older patients with cancer^27^ (eMethods in Supplement 1).

Our primary goal was to assess whether grade 3 or higher toxic effects were associated with a decline in QOL and/or physical functioning. However, many participants died during the first year; thus, a decrease in QOL and physical functioning could no longer occur due to mortality. Arguably, mortality can be considered the ultimate form of loss of QOL and physical functioning. Therefore, we designed a composite end point in which an unfavorable outcome was defined as either a decline in QOL and/or physical functioning or mortality. A decline in QOL was assessed with the EQ-5D-3L or EQ-VAS. Scores were compared with baseline scores using the minimal clinically important difference (EQ-5D-3L ≥0.06 or EQ-VAS ≥7 points^28^). A decline in physical functioning was defined as a decline in ADL (≥1-point increase or new institutionalization) or IADL (≥1-point decrease).^29^ The occurrence of grades 3 to 5 toxic effects, scored with the Common Terminology Criteria for Adverse Events, version 5^30^ up to 6 months after treatment initiation, was retrieved from the medical records. Secondary outcomes were dose reductions during treatment, early treatment withdrawal (inability to receive all planned cycles regardless of treatment duration), and unplanned hospitalizations within the first year (excluding scheduled admissions).

### Statistical Analysis

In prespecified analyses, participants were stratified by frailty. We used descriptive statistics using Mann-Whitney tests and χ^2^ tests to compare characteristics of patients with frailty and without frailty. The correlation between the number of impaired geriatric domains (range, 0-4) and the FI-CGA-10 (robust, prefrail, or frail) was tested using a Pearson correlation. Treatment outcomes and composite end points were stratified for frailty status and the number of impaired frailty domains.

Associations between grade 3 or higher toxic effects and a composite end point (a decline in QOL and/or physical functioning or mortality) were analyzed with univariable and multivariable logistic regression models. We calculated odds ratios (ORs) and 95% CIs. In patients with frailty, we also studied the association between toxic effects and mortality alone at 12 months. Possible confounding factors were predefined by creating a directed acyclic graph^31,32^ (eFigure 1 in Supplement 1). Confounding variables included in the model were age, treatment intent, and hospital. An up-front dose reduction was identified as an ancestor of the exposure rather than as a confounder. We performed additional analyses in a full model in which we added surgery, radiotherapy, and polychemotherapy to the original model. To prevent loss of information, missing follow-up questionnaires of living patients (n = 16) were coded as decline. As a sensitivity analysis, we repeated the analyses with missing questionnaires coded as no decline. We performed another sensitivity analysis in which we stratified the end point for treatment intent to investigate the role of predefined, palliative intent on treatment outcomes in patients with frailty. All analyses were performed using SPSS, version 25 (IBM Corp), and *P* < .05 was considered statically significant.

## Results

We included 276 older patients (eFigure 2 in Supplement 1); the median age was 74 years (IQR, 72-77 years); 99 patients (36%) were female, and 177 (64%) were male (Table 1). Of the total patients, 157 (57%) had gastrointestinal cancers, 196 (71%) received chemotherapy with curative intent, and 195 (71%) received polychemotherapy. Among all patients, 167 (61%) had a CCI of 1 or higher, and 145 patients (53%) had deficits in 2 or more of the 4 domains and were classified as frail. An impaired Geriatric 8 score was found in 68 patients (52%) without frailty and 113 patients (78%) with frailty. The number of impaired geriatric domains was strongly correlated with the FI-CGA-10 frailty index (Pearson correlation = 0.72).

**Table 1.** Baseline Characteristics

Variable	Participants, No. (%)			*P* value^b^
	Total (N = 276)	Without frailty (n = 131)	With frailty (n = 145)^a^	
Sex				
Female	99 (36)	38 (29)	61 (42)	.02
Male	177 (64)	93 (71)	86 (58)	
Age, median (IQR), y	74 (72-77)	74 (71-77)	75 (72-78)	.28
Tumor site				
Esophageal	88 (32)	48 (37)	40 (28)	.09
Colorectal	43 (16)	19 (15)	24 (17)	
Gynecologic	28 (10)	12 (9)	16 (11)	
Gastric cancer	26 (9)	17 (13)	9 (6)	
Genitourinary	24 (9)	12 (9)	12 (8)	
Lung	22 (8)	6 (5)	16 (11)	
Other^c^	45 (16)	17 (13)	28 (19)	
Treatment intent				
Curative	196 (71)	104 (79)	92 (63)	.004
Palliative	80 (29)	27 (21)	53 (37)	
Chemotherapy regimen^d^				
Monotherapy	81 (29)	30 (23)	51 (35)	.03
Polytherapy	195 (71)	101 (77)	94 (65)	
Starting dose				
Reduced up front	53 (19)	16 (12)	37 (26)	.006
Full dose	183 (66)	90 (69)	9 (64)	
Unknown	40 (15)	25 (19)	15 (10)	
No. of chemotherapy cycles, median (IQR)	5 (3-6)	5 (4-6)	5 (3-6)	.50
Surgery	104 (38)	61 (47)	43 (30)	.004
Concurrent radiotherapy	145 (53)	67 (51)	78 (54)	.66
Concurrent immunotherapy	5 (2)	1 (1)	4 (3)	.22
WHO status^e^				
0	83 (30)	41 (31)	42 (29)	.05
1	107 (39)	47 (36)	60 (41)	
≥2	18 (7)	4 (3)	14 (10)	
Unknown	68 (25)	39 (30)	29 (20)	
CCI comorbidity^f^				
0	109 (40)	68 (52)	41 (28)	NA
1	74 (27)	28 (21)	46 (32)	
2	39 (14)	13 (10)	26 (18)	
≥3	54 (20)	22 (17)	32 (22)	
Polypharmacy^g^	165 (60)	62 (47)	103 (71)	NA
Home care	22 (8)	4 (3)	18 (12)	NA
Delirium in past months	7 (3)	0	7 (5)	NA
Falls in last 6 mo				
No	227 (82)	128 (98)	99 (68)	NA
Yes	49 (18)	3 (2)	46 (32)	
Geriatric 8^h^				
Normal	75 (27)	50 (38)	25 (17)	NA
Impaired	181 (66)	68 (52)	113 (78)	
Unknown	20 (7)	13 (10)	7 (5)	
6CIT^h^				
Normal	239 (87)	127 (97)	112 (77)	NA
Impaired	29 (11)	1 (1)	28 (19)	
Unknown	8 (3)	3 (2)	5 (3)	
Mini Nutritional Assessment^h^				
Well nourished	103 (37)	65 (50)	38 (26)	NA
Risk of malnutrition	169 (61)	62 (47)	107 (74)	
Unknown	4 (1)	4 (3)	0	
PHQ-2^h^				
Normal	195 (71)	86 (66)	109 (75)	NA
Abnormal	23 (8)	6 (5)	17 (12)	
Unknown	58 (20)	39 (30)	19 (13)	
Activities of daily living^h^				
Independent	271 (99)	130 (48)	141 (52)	NA
Dependent	3 (1)	0	3 (1)	
Unknown	2 (1)	1 (1)	1 (1)	
Instrumental activities of daily living^h^				
Independent	214 (78)	126 (59)	88 (41)	NA
Dependent	56 (20)	0	56 (39)	
Unknown	6 (2)	5 (4)	1 (1)	

Abbreviations: 6CIT, Six-Item Cognitive Impairment Test; CCI, Charlson Comorbidity Index; PHQ-2, Patient Health Questionnaire-2; WHO, World Health Organization.

^a^ Frailty was defined as having 2 or more impaired geriatric domains.

^b^ Not applicable (NA) indicates that all geriatric variables that were used to define patients with and without frailty were not tested for statistical differences between patients with and without frailty, since stratification for frailty was based on these variables.

^c^ Other tumor types included pancreatic cancer, breast cancer, liver cancer, anal cancer, lymphoma, myeloid leukemia, and head and neck cancer.

^d^ The most commonly prescribed chemotherapy regimens were carboplatin or paclitaxel (n = 99), a fluoropyrimidine combined with oxaliplatin (n = 48), fluoropyrimidine monotherapy (n = 36), other carboplatin-based chemotherapy (n = 26), cisplatin-based chemotherapy (n = 21), and docetaxel monotherapy (n = 18).

^e^ Indicates the patient’s general condition. Numbers range from 0 to 4, with higher numbers representing a poorer general condition.

^f^ Defined as CCI ≥1.

^g^ Defined as 5 or more medicines.

^h^ Functional status categories are described in the Methods section.

**Figure 1.**
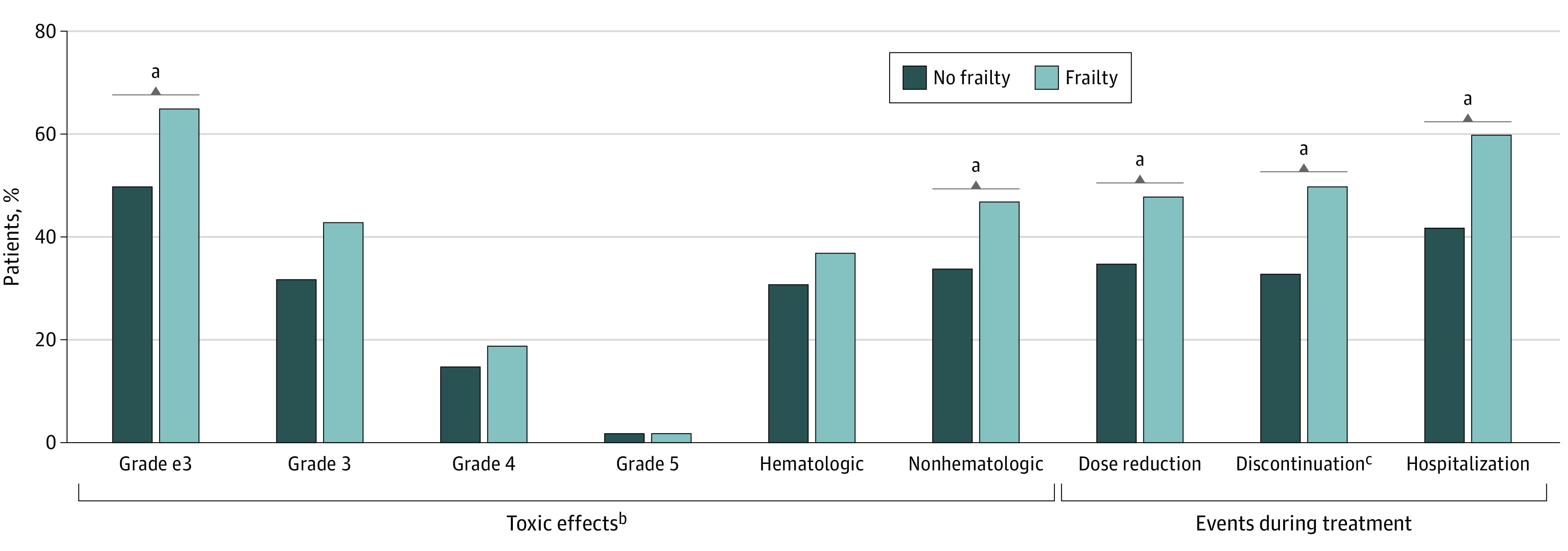
Incidence of Various Treatment Outcomes in All Patients by Frailty Status Frequencies of grades 3 to 5 toxic effects (*P* = .01), nonhematologic toxic effects (*P* = .03), dose reduction during treatment (*P* = .04), early treatment discontinuation (*P* = .009), and hospitalization (*P* = .003) were significantly different between patients with and without frailty, analyzed with a χ^2^ test. ^a^*P* < .05. ^b^The worst grade of toxic effects was documented (grade e3). Of the patients who developed grades 3 to 5 toxic effects, 91% developed their first toxic effects within the first 2 months after chemotherapy initiation, and 9% developed their first toxic effects after 2 months. ^c^Among patients with frailty, reasons for early treatment discontinuation were toxic effects (64%), tumor progression (29%), death to other cause (3%), clinical deterioration (3%), or other reasons (1%). Among patients without frailty, reasons for early treatment discontinuation were toxic effects (56%), tumor progression (28%), or clinical deterioration (16%).

In total, 160 patients (58%) developed grades 3 or higher toxic effects, of whom 146 (91%) developed their first toxic effects within the first 2 months after chemotherapy initiation. Of patients with frailty, 94 (65%) developed grade 3 or higher toxic effects (Figure 1). Seventy patients (48%) had a dose reduction during treatment, 73 (50%) discontinued treatment early, and 87 (60%) were hospitalized in the first year, of whom 71 (82%) were hospitalized during chemotherapy treatment.

Of patients without frailty, 66 (50%) developed grade 3 or higher chemotherapy-related toxic effects, 46 (35%) had a dose reduction during treatment, 43 (33%) discontinued treatment, and 55 (42%) were hospitalized in the first year, of whom 37 (67%) were hospitalized during chemotherapy treatment. Rates of grade 3 or higher toxic effects (65% vs 50%; *P* = .01), nonhematologic toxic effects (47% vs 34%; *P* = .03), dose reduction (48% vs 35%; *P* = .04), early discontinuation (50% vs 33%; *P* = .009), and hospitalization (60% vs 42%; *P* = .003) differed significantly between patients with frailty and those without (Figure 1).

**Figure 2.**
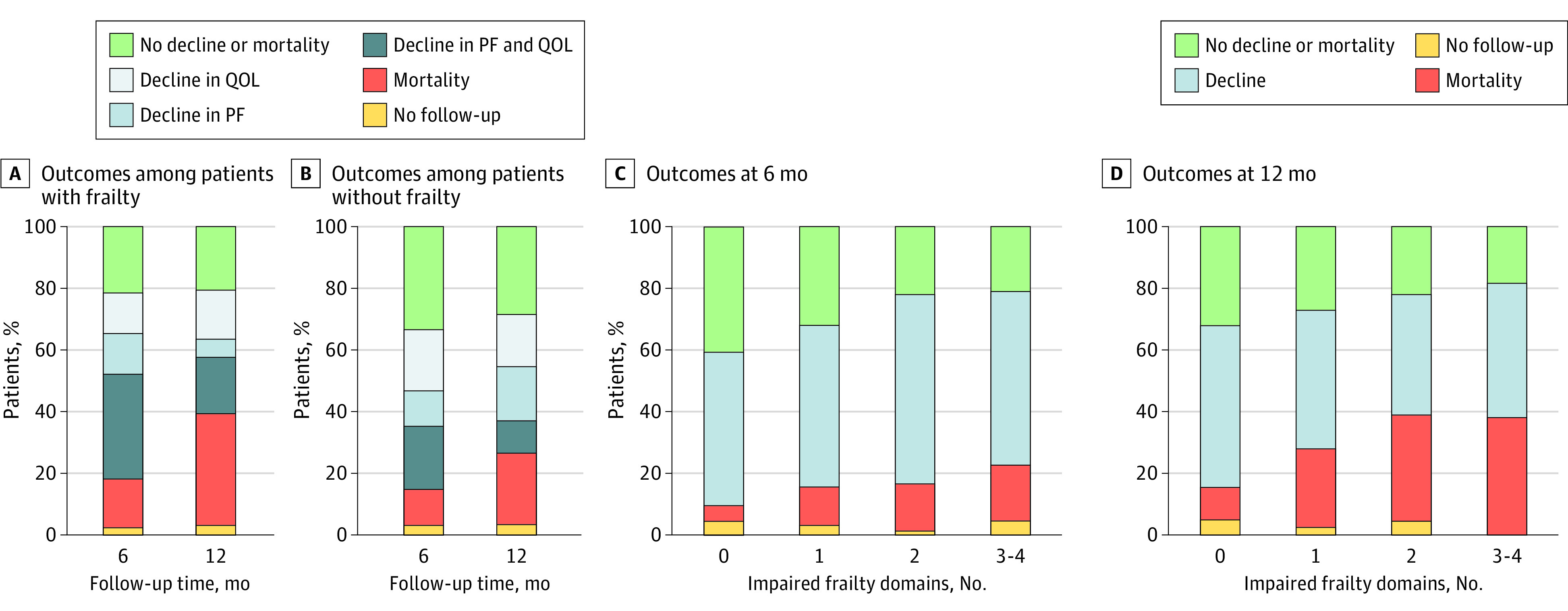
Composite End Points After 6 Months and 12 Months by Frailty Status and by the Absolute Number of Impaired Frailty Domains A, Among living patients with frailty at 6 months, 40% still received chemotherapy, and 60% either completed or discontinued treatment. Among living patients with frailty at 12 months, 17% still received chemotherapy, and 83% either completed or discontinued treatment. B, Among living patients without frailty at 6 months, 30% still received chemotherapy, and 70% either completed or discontinued chemotherapy. Among living patients without frailty at 12 months, 9% still received chemotherapy, and 91% completed or discontinued treatment. C and D, Frailty domains include somatic, functional, psychological, and social. Decline represents either a decline in quality of life (QOL) or a decline in physical functioning (PF). No follow-up consists of living patients without a completed follow-up questionnaire at the specified time point.

Of patients with frailty, 30 (21%) reported a preserved QOL and physical functioning after 6 months and 110 (76%) had a poor outcome: 87 (60%) experienced a decline and 23 (16%) died (Figure 2A). After 12 months, 29 (20%) had a preserved QOL and physical functioning, while 110 (76%) had a poor outcome: 58 (40%) experienced a decline and 52 (36%) died. Of the patients without frailty, 43 (33%) had a preserved QOL and physical functioning after 6 months, while 84 (64%) had a poor outcome: 68 (52%) experienced a decline in QOL and/or physical functioning and 16 (12%) died (Figure 2B). After 12 months, 37 (28%) had a preserved QOL and physical functioning, while 89 (68%) had a poor outcome: 59 (45%) reported a decline in QOL and/or physical functioning, and 30 (23%) died. After stratifying results by the number of impaired frailty domains, the percentage of patients having a poor outcome at 6 months increased from 55% in patients without impaired domains to 74% in patients with 3 to 4 impaired domains (Figure 2C). Similarly, after 12 months, the percentage of patients with poor outcomes increased per number of impaired frailty domains (Figure 2D).

**Figure 3.**
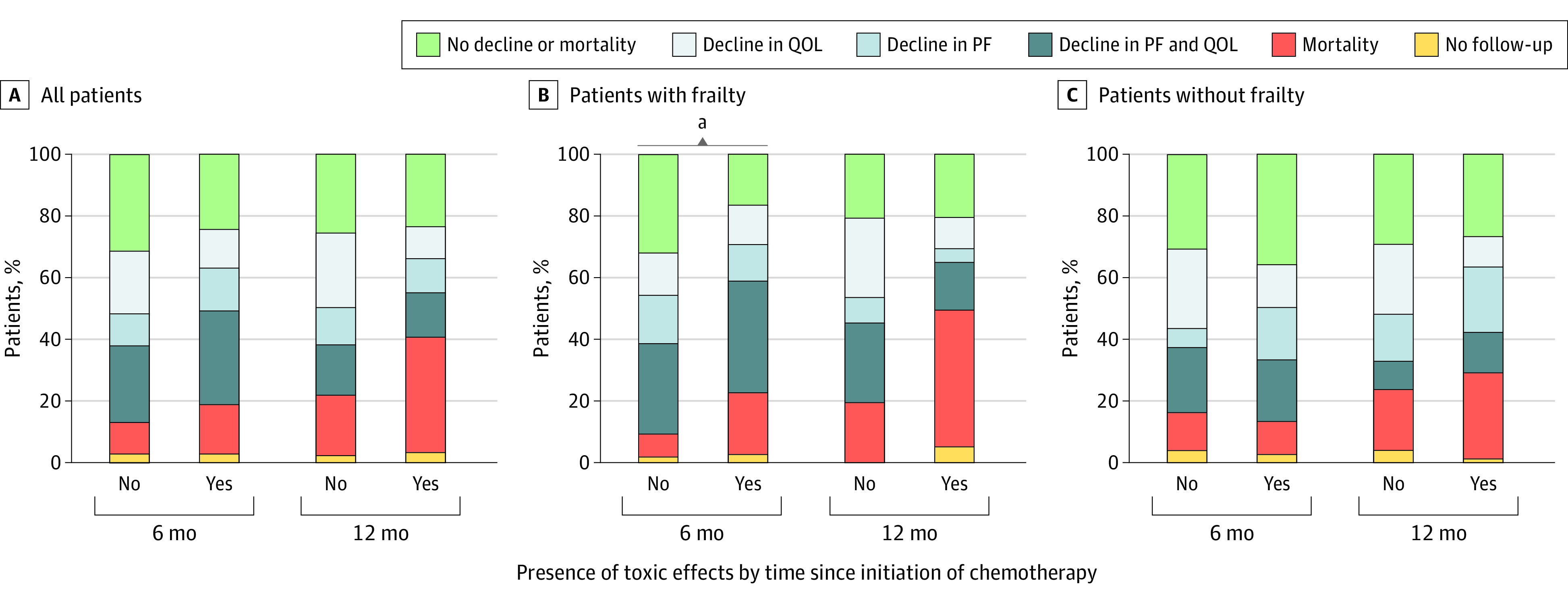
Composite End Points of Patients After 6 Months and 12 Months by Grade 3 or Higher Chemotherapy-Related Toxic Effects Status No follow-up consists of living patients without a completed follow-up questionnaire at the specified time point. PF indicates physical functioning; QOL, quality of life. ^a^*P* < .05, derived from the multivariable logistic regression models shown in Table 2.

In all patients, composite end points at 6 months were comparable for individuals with and without toxic effects (Figure 3). Grade 3 or higher toxic effects were not associated with the composite end point at 6 months (OR, 1.47; 95% CI, 0.85-2.55) (Table 2). After 12 months, more patients with toxic effects died, but toxic effects were not associated with the composite end point (OR, 1.08; 95% CI, 0.60-1.94).

**Table 2.** Association Between Grade 3 or Higher Toxic Effects and Unfavorable Outcomes at 6 Months and 12 Months^a^

Grade ≥3 toxic effects by time since initiation of chemotherapy	Univariable logistic regression		Multivariable logistic regression^b^	
	Odds ratio (95% CI)	*P* value	Odds ratio (95% CI)	*P* value
All patients				
6 mo	1.42 (0.83-2.42)	.20	1.47 (0.85-2.55)	.17
12 mo	1.08 (0.62-1.90)	.78	1.08 (0.60-1.94)	.80
Patients with frailty				
6 mo	2.41 (1.07-5.41)	.03	2.62 (1.14-6.05)	.02
12 mo	1.00 (0.42-2.37)	1.00	1.09 (0.45-2.64)	.86
Patients without frailty				
6 mo	0.79 (0.38-1.65)	.54	0.76 (0.36-1.64)	.49
12 mo	1.05 (0.48-2.27)	.91	1.06 (0.46-2.43)	.90

^a^ Unfavorable outcomes include decline in quality of life and/or physical functioning or mortality.

^b^ Multivariable logistic regression models were adjusted for age, hospital type, and treatment intent. Toxic effects, treatment intent, and hospital were added to the model as binary variables and age was added as a continuous variable.

After stratification for frailty, the percentage of patients with frailty with preserved QOL and physical functioning after 6 months was lower in those with toxic effects (16%) compared with those without toxic effects (31%). Moreover, 19 patients (20%) among those with frailty and toxic effects died, whereas only 4 (8%) of those without toxic effects died. The multivariable logistic regression model showed that toxic effects were associated with an unfavorable outcome (OR, 2.62; 95% CI, 1.14-6.05) (Table 2 and eTable 1 in Supplement 1). Composite end points after 12 months also differed between those with and without toxic effects, as 42 (45%) of patients with toxic effects died compared with 10 (20%) without toxic effects. There was no association between toxic effects and an unfavorable outcome after 12 months in the multivariable analysis (OR, 1.09; 95% CI, 0.45-2.64). As these results might be biased by the high mortality rate of patients with toxic effects, we also analyzed the association between toxic effects and mortality and found a significant association between toxic effects and mortality after 12 months (OR 3.54; 95% CI, 1.50-8.33) (eTable 2 in Supplement 1).

In patients without frailty, composite end points after 6 months were similar for those with and without grade 3 or higher toxic effects. In a multivariable logistic regression, toxic effects were not associated with an unfavorable outcome after 6 months (OR, 0.76; 95% CI, 0.36-1.64) (Table 2 and eTable 3 in Supplement 1). After 12 months, composite end points were also similar for those who had toxic effects and those who did not. Grade 3 or higher toxic effects were not associated with an unfavorable outcome in a multivariable analysis (OR, 1.06; 95% CI, 0.46-2.43).

Using sensitivity analyses, we first investigated the association of predefined treatment intent with outcomes among patients with frailty (eFigure 3 in Supplement 1). High rates of a decline in QOL and/or physical functioning or mortality were seen both in those treated with palliative intent and in those treated with curative intent, illustrating that outcomes of patients with frailty were poor irrespective of treatment intent. Second, to assess whether the results of the logistic regression models were different if missing questionnaires were defined as no decline instead of decline, we performed a sensitivity analysis and found that results did not differ (eTable 4 in Supplement 1). Third, we added various treatment characteristics to the original model and found that, in the full model, toxic effects remained associated with an unfavorable outcome after 6 months in patients with frailty (eTable 5 in Supplement 1).

## Discussion

This study is the first, to our knowledge, to investigate the association between grade 3 or higher chemotherapy-related toxic effects and a decline in QOL and/or physical functioning or mortality in older patients with cancer. Irrespective of frailty status and treatment intent, most patients had either a decline in QOL and/or physical functioning or died after 6 months and 12 months. Among patients with frailty, 65% experienced grade 3 or higher toxic effects, and grade 3 or higher toxic effects were associated with a decline in QOL and/or physical functioning or mortality. Grade 3 or higher toxic effects were not associated with unfavorable outcomes in older patients without frailty.

The percentages of patients with a decline in QOL and physical functioning in our study were slightly higher than in previous studies. Kenis et al^33^ investigated physical functioning 2 to 3 months after chemotherapy initiation in a prospective cohort (N = 439) and showed that functional decline occurred in one-third of older patients with various tumor types. Hoppe and colleagues^34^ found that 17% of patients reported a functional decline in a study with 364 older patients receiving first-line chemotherapy for various tumor types. Of our study’s participants, 157 (57%) had upper gastrointestinal cancers, which are generally tumor types with a poor prognosis. Additionally, 145 (53%) of our study’s participants lived with frailty and therefore may have had an increased risk of decreased QOL, irrespective of treatment.^35^ These factors may explain the higher percentages of a decline in QOL and physical functioning in our cohort.

Of patients with frailty, 65% developed grade 3 or higher toxic effects, 50% discontinued treatment early, and 60% were hospitalized, which aligns with previous literature.^36,37,38^ Not only did patients with frailty have an increased risk of toxic effects, but the occurrence of toxic effects was also associated with poor long-term outcomes. To date, few studies have investigated the association between toxic effects and long-term outcomes in older patients receiving chemotherapy. A prospective pilot study in older patients (N = 37) also found an association between grade 3 or higher chemotherapy toxic effects and physical functioning or mental health at the end of treatment.^39^ Preliminary secondary analyses from the Geriatric Assessment Intervention for Reducing Toxicity in Older Patients With Advanced Cancer trial demonstrated that older patients with patient-reported grade 3 or higher toxic effects were more likely to experience functional decline within 6 months as well.^40^ The association between toxic effects and long-term outcomes may be because toxic effects and hospitalizations may lead to accelerated functional decline. Additionally, the high rates of toxic effects in patients with frailty may result in early treatment discontinuation, possibly leading to disease progression and poor outcomes associated with the cancer itself. Last, those at risk of developing toxic effects may also be the patients with the worst baseline characteristics who are vulnerable to poor outcomes, irrespective of treatment.^35^ Decline after 12 months may be influenced by multiple factors, with disease progression possibly contributing more than toxic effects do.

In our study, the high rates of grade 3 or higher toxic effects, unplanned hospitalizations, and functional decline among patients with frailty, in both those treated with curative and palliative chemotherapy, imply that treatment goals are not met in the vast majority of this population. This raises the question of whether to use chemotherapy to treat older patients with frailty. However, patients with frailty who do not receive anticancer treatment may also decline. Moreover, chemotherapy may improve symptoms and thus may improve QOL. Personalized treatment with adapted treatment and intensified support trajectories should, however, be required, along with identification of patients at high risk of poor outcomes in whom chemotherapy may be contraindicated. Since an oncologist’s judgment in predicting toxic effects may be limited,^41,42,43^ pretreatment frailty screening, followed by a CGA, can be used to identify those at risk for poor treatment outcomes. A CGA is a multidimensional process to identify medical, social, and functional needs^44^ and is associated with chemotherapy-related toxic effects, functional decline, and mortality.^34,37,45^ By performing a pretreatment CGA, physicians may facilitate nononcologic interventions or individualize treatment plans, thereby likely reducing the risk of toxic effects and possibly improving QOL.^46^ Previous trials showed that a GA-based intervention could reduce chemotherapy-related toxic effects and early treatment discontinuation compared with usual care.^47,48^

Another possibility to improve treatment tolerability in a selected group of patients is to adapt treatment plans, such as performing up-front dose reduction or prescribing less-toxic chemotherapy regimens.^49^ Previous trials in older patients demonstrated that up-front dose-reduced chemotherapy led to fewer toxic effects and improved QOL, without compromising survival.^50,51,52^ Individualizing treatment plans based on frailty status might therefore be a promising solution to improve outcomes. Unfortunately, some patients with frailty do not benefit from chemotherapy. Although omitting treatment in these patients may lead to premature death, best supportive care can still be the preferred option. Taking into account aging populations, rising health care costs, and an anticipated shortage of health care workers worldwide,^53^ it will be important to identify those who may not benefit from chemotherapy in the coming years.

### Strengths and Limitations

TENT is a unique, multicenter cohort that includes a large number of older patients with and without frailty. Recruiting older patients with frailty for clinical studies comes with many challenges, such as difficulties with traveling to the research site, other medical conditions, or lack of social support.^54^ By conducting follow-up questionnaires by telephone rather than in person, we kept the participation burden minimal, which might have resulted in a large number of patients with frailty willing to participate. Consequently, the outcomes of this study are relevant for older patients seen in daily practice and can be extrapolated to the general older population. Additional strengths are the high rates of completed follow-up, which may reduce bias, and relatively large sample size.

This study also has limitations. Although we made every effort to identify possible confounders and evaluated the robustness of our results in different models, results could still have been subject to unidentified or unmeasured confounders. Additionally, the OR tends to overestimate the risk estimate of having the outcome in the exposed group when the incidence of the outcome is high, so it should be interpreted with caution. Furthermore, even among patients without frailty, 68 (52%) had an impaired Geriatric 8 score, suggesting that vital older patients may be underrepresented in our study. It should also be noted that we measured only grades 3 to 5 toxic effects, while previous studies have shown that low-grade or patient-reported toxic effects may also influence treatment outcomes in older patients.^55,56^ In addition, while the findings showed that 91% of patients with toxic effects developed their first toxic effects within 2 months, the exact date of the registered toxic effect was unknown. Last, the modest sample size of the subgroup analyses may have resulted in wide CIs. While our study’s population was heterogeneous with various tumor types, it reflected a clinical setting’s practice and demonstrated that generalizability of positive outcomes observed in oncology trials is limited when applied to a more diverse, general patient population.^57^ To further support these findings, future studies should focus on specific tumor or treatment types.

## Conclusions

In this cohort study, frailty was not only associated with grade 3 or higher chemotherapy-related toxic effects, but the occurrence of toxic effects among patients with frailty was also associated with a decline in QOL and/or physical functioning or mortality at 6 and 12 months. Grade 3 or higher chemotherapy-related toxic effects were not associated with poor outcomes in patients without frailty. Geriatric assessment–driven interventions and individualized treatment adaptions could prevent a treatment-related decline of remaining health.

## Notes

Supplement 1.
eMethods.
**eFigure 1.** Directed Acyclic Graph (DAG) **eFigure 2.** Flow Diagram of Included Patients **eFigure 3.** Composite End Points After 6 and 12 Months in Patient With Frailty, Stratified by Treatment Intent **eTable 1.** Association of Grade ≥3 Toxicity and Unfavorable Outcomes in Frail, Older Patients, Including Point Estimates of Confounding Factors **eTable 2.** Association of Grade ≥3 Toxicity and Mortality After 12 Months in Frail, Older Patients **eTable 3.** Association of Grade ≥3 Toxicity and Unfavorable Outcomes in Nonfrail, Older Patients, Including Point Estimates of Confounding Factors **eTable 4.** Sensitivity Analysis: Association of Grade ≥3 Toxicity and Unfavorable Outcomes With Different Coding of Missing Questionnaires **eTable 5.** Sensitivity Analysis: Association of Grade ≥3 Toxicity and Unfavorable Outcomes in the ‘Full Model’ With Both Confounders and Treatment Characteristics
eReferences

Supplement 2.
Data Sharing Statement
