# Supplementary material for: Chemotherapy-Related Toxic Effects and Quality of Life and Physical Functioning in Older Patients
Source: JAMA Netw Open. 2023 Oct 23;6(10):e2339116. doi: 10.1001/jamanetworkopen.2023.39116 (PMC10594146; doi:10.1001/jamanetworkopen.2023.39116)
Supplement: Supplement 1. — eMethods. eFigure 1. Directed Acyclic Graph (DAG) eFigure 2. Flow Diagram of Included Patients eFigure 3. Composite End Points After 6 and 12 Months in Patient With Frailty, Stratified by Treatment Intent eTable 1. Association of Grade ≥3 Toxicity and Unfavorable Outcomes in Frail, Older Patients, Including Point Estimates of Confounding Factors eTable 2. Association of Grade ≥3 Toxicity and Mortality After 12 Months in Frail, Older Patients eTable 3. Association of Grade ≥3 Toxicity and Unfavorable Outcomes in Nonfrail, Older Patients, Including Point Estimates of Confounding Factors eTable 4. Sensitivity Analysis: Association of Grade ≥3 Toxicity and Unfavorable Outcomes With Different Coding of Missing Questionnaires eTable 5. Sensitivity Analysis: Association of Grade ≥3 Toxicity and Unfavorable Outcomes in the ‘Full Model’ With Both Confounders and Treatment Characteristics eReferences [file jamanetwopen-e2339116-s001.pdf]

Supplemental Online Content

Baltussen JC, de Glas NA, van Holstein Y, et al. Chemotherapy-related toxic effects and quality of life and physical functioning in older patients. *JAMA Netw Open*. 2023;6(10):e2339116. doi:10.1001/jamanetworkopen.2023.39116

eMethods
eFigure 1. Directed Acyclic Graph (DAG)
eFigure 2. Flow Diagram of Included Patients
eFigure 3. Composite End Points After 6 and 12 Months in Patient With Frailty, Stratified by Treatment Intent
eTable 1. Association of Grade ≥3 Toxicity and Unfavorable Outcomes in Frail, Older Patients, Including Point Estimates of Confounding Factors
eTable 2. Association of Grade ≥3 Toxicity and Mortality After 12 Months in Frail, Older Patients
eTable 3. Association of Grade ≥3 Toxicity and Unfavorable Outcomes in Nonfrail, Older Patients, Including Point Estimates of Confounding Factors
eTable 4. Sensitivity Analysis: Association of Grade ≥3 Toxicity and Unfavorable Outcomes With Different Coding of Missing Questionnaires
eTable 5. Sensitivity Analysis: Association of Grade ≥3 Toxicity and Unfavorable Outcomes in the 'Full Model' With Both Confounders and Treatment Characteristics
eReferences

This supplemental material has been provided by the authors to give readers additional information about their work.

© 2023 Baltussen JC et al. *JAMA Network Open*.

eMethods

To validate the definition of frailty we used based on the 4 geriatric domains, we calculated a cumulative frailty deficit index, using the a 10-Item Frailty Index Based on a Comprehensive Geriatric Assessment (FI-CGA-10) developed by Rockwood and colleagues[1]. This frailty tool is developed and validated in an older population with cancer[2]. Using the same definition as used by Nishijama et al.[2], we calculated the cumulative frailty deficit index based on cognition (impaired 6-CIT, previous delirium or diagnosis dementia), mood (impaired PHQ-2), mobility (impaired gait speed or inability to walk one block), balance (falls in the past 6 months), nutrition (impaired MNA), basic and instrumental activities of daily living (impaired Katz-ADL or impaired Lawton-IADL), social support (living alone), and comorbidity (CCI). Since we did not gather information on vision or hearing for most patients, we did not include the ‘communication’ domain in the Index and divided the scores by 9 instead of 10. Scores of the FI-CGA-10 range from 0 to 1 and categorizes patients in robust (0-0.19), pre-frail (0.2-0.35) or frail (≥0.35) patients.

eMethods: Calculation of FI-CGA-10

**9 domains**	0 points	0.5 points	1 point
**Cognition**	6-CIT 0-6 points	6-CIT 8-9 points	Previous delirium, diagnosis of dementia or 6-CIT of 10-28 points
**Mood**	Normal PHQ-2		Impaired PHQ-2
**Mobility**	Gait speed ≥1.0 m/s. If walking test not done: ability to walk one block	0.8 m/s ≤ Gait speed <1.0 m/s	Gait speed <0.8 m/s. If walking test not done: inability to walk one block
**Balance**	No falls in past 6 months		Falls in past 6 month
**Nutrition**	Not impaired based on the MNA	At risk of malnutrition based on the MNA	Malnourished based on the MNA
**ADL**	Not impaired	ADL impaired, only due to incontinence question	ADL impaired, including at least one impairment other than incontinence
**IADL**	Not impaired		Impaired
**Social support**	Living with others		Living alone
**Comorbidity**	0 (based on CCI)	1-2 (based on CCI)	3 or more (based on CCI)

© 2023 Baltussen JC et al. *JAMA Network Open*.

eFigure 1. Directed Acyclic Graph (DAG)

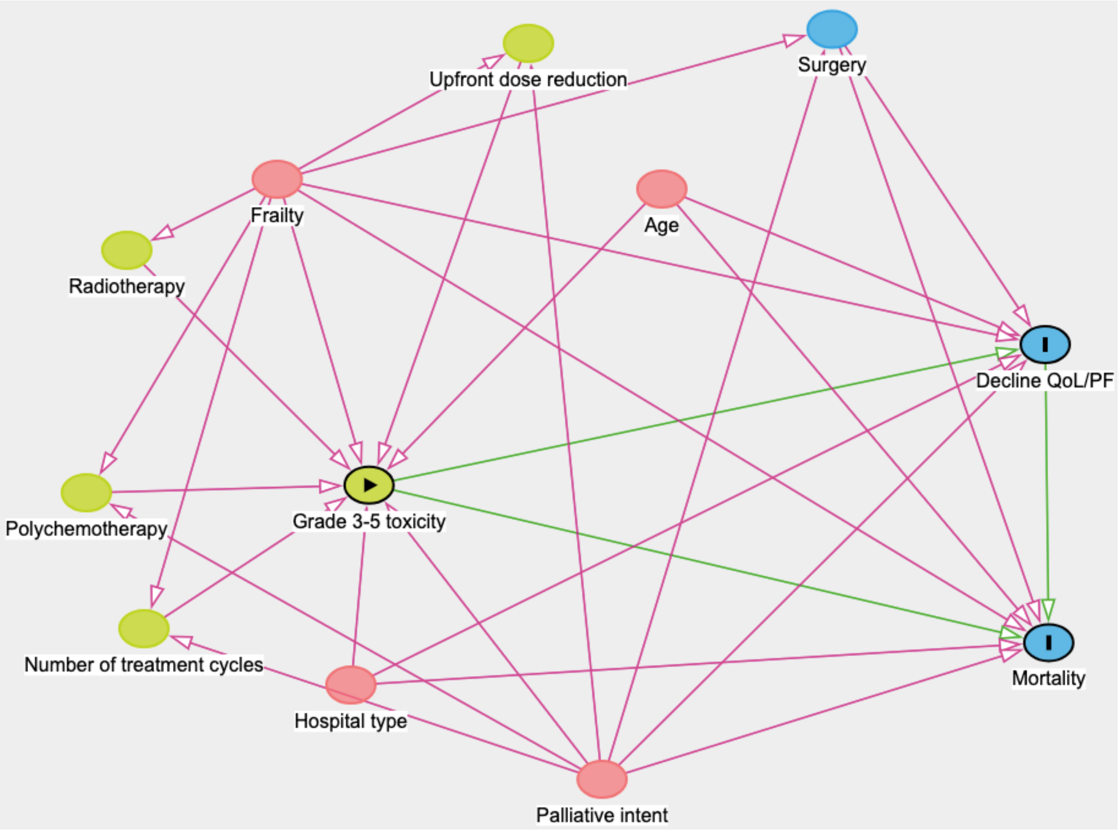

The DAG was created by using DAGitty, a browser-based environment for creating, editing, and analyzing causal diagrams (also known as directed acyclic graphs or causal Bayesian networks). The DAG shows the primary outcome (decline, mortality), primary exposure (grade 3-5 toxicity) and potential confounders or effect modifiers (age, frailty, hospital type and palliative intent). The figure also illustrates other covariables, which are ancestors of the exposure (radiotherapy, upfront dose reduction, polychemotherapy and number of treatment cycles). Green path is a causal path, pink path is a biasing path.

© 2023 Baltussen JC et al. *JAMA Network Open*.

eFigure 2. Flow Diagram of Included Patients

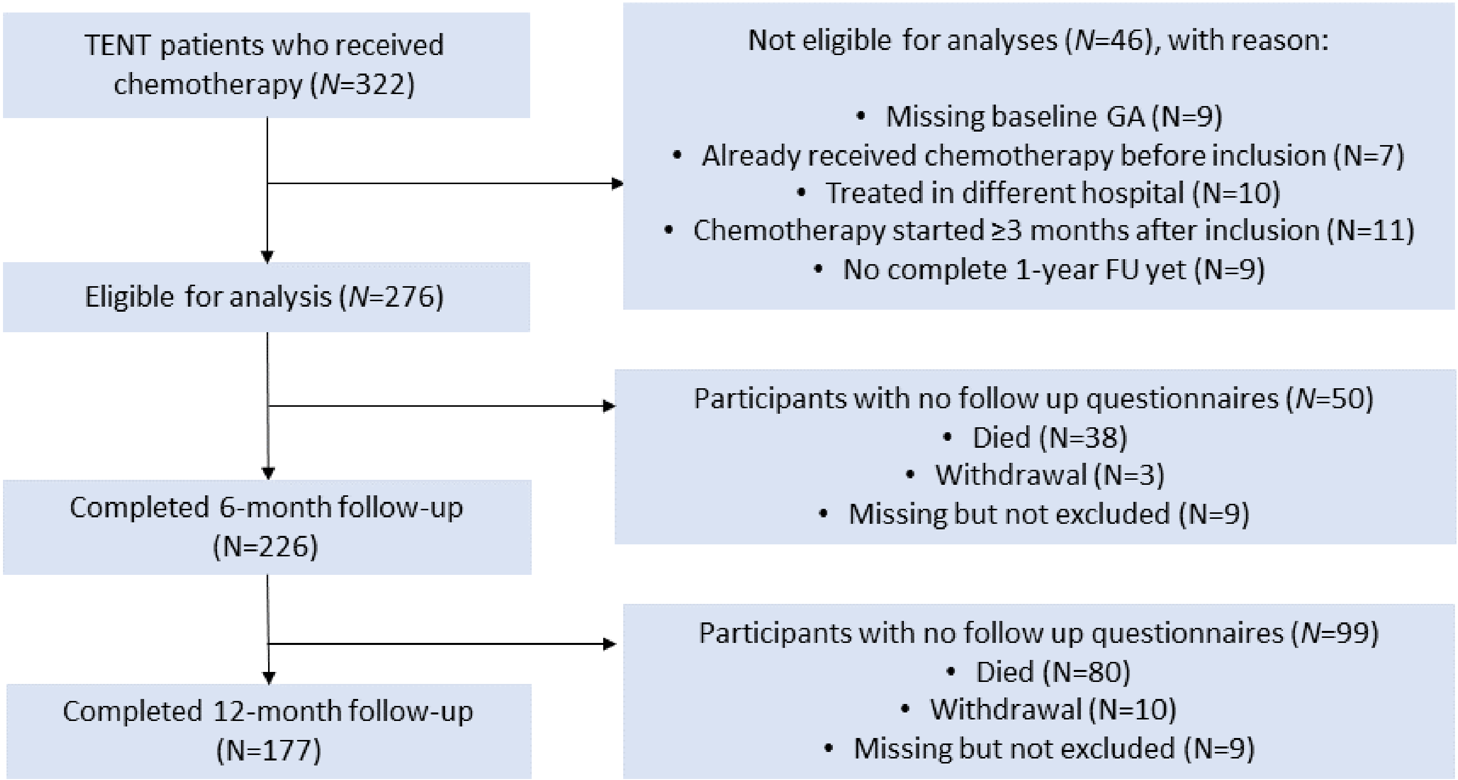

© 2023 Baltussen JC et al. *JAMA Network Open*.

eFigure 3. Composite End Points After 6 and 12 Months in Patient With Frailty, Stratified by Treatment Intent

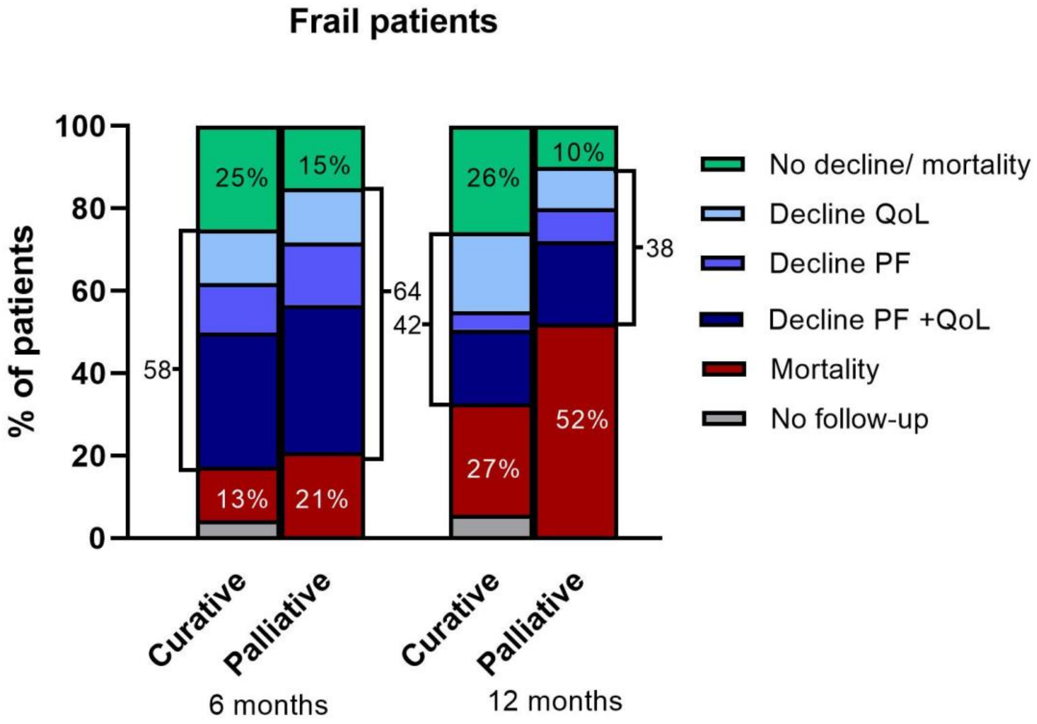

'No follow-up' group consists of alive patients without complete follow-up questionnaire at timepoint. Abbreviations: PF; physical functioning, QoL; quality of life.

© 2023 Baltussen JC et al. *JAMA Network Open*.

eTable 1. Association of Grade ≥3 Toxicity and Unfavorable Outcomes in Frail, Older Patients, Including Point Estimates of Confounding Factors

	**Univariable**		**Multivariable**	
**6 months**	*OR (95% CI)*	*P-value*	*OR (95% CI)*	*P-value*
**Severe toxicity**	2.41 (1.07 - 5.41)	.03	2.62 (1.14 – 6.05)	.02
**Palliative intent**	1.88 (0.77 - 4.56)	.17	2.34 (0.90 – 6.06)	.08
**Age**	1.03 (0.94 - 1.14)	.52	1.04 (0.94 - 1.15)	.49
**Hospital 1**	Ref		Ref	
**Hospital 2**	1.19 (0.24 - 6.03)	.83	1.11 (0.20 - 5.94)	.90
**Hospital 3**	0.58 (0.25 - 1.34)	.20	0.48 (0.21 - 1.17)	.11
**12 months**	*OR (95% CI)*	*P-value*	*OR (95% CI)*	*P-value*
**Severe toxicity**	1.00 (0.42 - 2.37)	1.00	1.09 (0.45 - 2.64)	.86
**Palliative intent**	3.21 (1.14 – 9.05)	.03	3.34 (1.15 – 9.70)	.03
**Age**	1.03 (0.93 - 1.14)	.57	1.02 (0.92 – 1.13)	.70
**Hospital 1**	Ref		Ref	
**Hospital 2**	0.75 (0.18 - 3.14)	.69	0.63 (0.14 – 2.75)	.53
**Hospital 3**	1.07 (0.44 - 2.58)	.88	0.83 (0.33 – 2.08)	.69

Uni- and multivariable logistic regression analysis on factors associated with an unfavorable outcome (either decline in QoL/physical functioning or mortality) in frail older patients, also showing the risk estimates of the confounding factors. Toxicity, treatment intent and hospital were added to the model as binary variables and age as continuous variable. Abbreviations; CI: confidence interval, OR: odds ratio.

© 2023 Baltussen JC et al. *JAMA Network Open*.

eTable 2. Association of Grade ≥3 Toxicity and Mortality After 12 Months in Frail, Older Patients

	Univariable		Multivariable*	
	OR (95% CI)	*P-value*	*OR (95% CI)*	*P-value*
**Grade ≥3 toxicity**	3.04 (1.36 – 6.78)	.007	3.54 (1.50 - 8.33)	.004

Uni- and multivariable logistic regression analysis on factors associated with mortality after 12 months in frail older patients.
*Multivariable logistic regression models were adjusted for age, hospital type and treatment intent. Toxicity, treatment intent and hospital were added to the model as binary variables and age as continuous variable.

© 2023 Baltussen JC et al. *JAMA Network Open*.

eTable 3. Association of Grade ≥3 Toxicity and Unfavorable Outcomes in Nonfrail, Older Patients, Including Point Estimates of Confounding Factors

	Univariable		Multivariable	
**6 months**	*OR (95% CI)*	*P-value*	*OR (95% CI)*	*P-value*
**Severe toxicity**	0.79 (0.38 - 1.65)	.54	0.76 (0.36 - 1.64)	.49
**Palliative intent**	1.93 (0.72 - 5.21)	.19	1.70 (0.61 – 4.73)	.31
**Age**	1.05 (0.95 - 1.16)	.34	1.03 (0.93 – 1.11)	.58
**Hospital 1**	Ref		Ref	
**Hospital 2**	0.48 (0.16 - 1.39)	.18	0.48 (0.16 - 1.44)	.19
**Hospital 3**	1.51 (0.54 - 4.17)	.43	1.30 (0.46 – 3.74)	.62
**12 months**	*OR (95% CI)*	*P-value*	*OR (95% CI)*	*P-value*
**Severe toxicity**	1.05 (0.48 - 2.27)	.91	1.06 (0.46 – 2.43)	.90
**Palliative intent**	6.09 (1.36 - 27.30)	.02	5.65 (1.22 – 26.14)	.03
**Age**	1.09 (0.97 - 1.21)	.14	1.07 (0.96 – 1.20)	.24
**Hospital 1**	Ref		Ref	
**Hospital 2**	0.37 (0.12 - 1.16)	.09	0.45 (0.14 - 1.53)	.20
**Hospital 3**	1.40 (0.47 - 4.18)	.54	0.94 (0.30 - 3.02)	.92

Uni- and multivariable logistic regression analysis on factors associated with an unfavorable outcome (either decline in QoL/physical functioning or mortality) in non-frail older patients, also showing the risk estimates of the confounding factors. Toxicity, treatment intent and hospital were added to the model as binary variables and age as continuous variable. Abbreviations; CI: confidence interval, OR: odds ratio.

© 2023 Baltussen JC et al. *JAMA Network Open*.

eTable 4. Sensitivity Analysis: Association of Grade ≥3 Toxicity and Unfavorable With Different Coding of Missing Questionnaires

	Multivariable*, 6 months		Multivariable*, 12 months	
**Frail**	*OR (95% CI)*	*P-value*	*OR (95% CI)*	*P-value*
Grade ≥3 toxicity	2.33 (1.04 – 5.24)	.04	0.77 (0.32 – 1.85)	.55
**Non-frail**	*OR (95% CI)*	*P-value*	*OR (95% CI)*	*P-value*
Grade ≥3 toxicity	0.78 (0.37 – 1.64)	.51	1.24 (0.54 – 2.82)	.62

Sensitivity analysis of multivariable logistic regression with different coding of missing questionnaires. In the original logistic regression model, missing follow-up questionnaires of alive patients (N=16) were coded as 'decline'. For this sensitivity analysis, we repeated the analyses with missing questionnaires coded as 'no decline'. Multivariable logistic regression models were adjusted for age, hospital type and treatment intent.

eTable 5. Sensitivity Analysis: Association of Grade ≥3 Toxicity and Unfavorable Outcomes in the 'Full Model' With Both Confounders and Treatment Characteristics

	Multivariable, 6 months		Multivariable, 12 months	
**Frail**	*OR (95% CI)*	*P-value*	*OR (95% CI)*	*P-value*
Grade ≥3 toxicity	3.03 (1.24 – 7.35)	.02	1.07 (0.42 – 2.74)	.90
**Non-frail**	*OR (95% CI)*	*P-value*	*OR (95% CI)*	*P-value*
Grade ≥3 toxicity	0.78 (0.36 – 1.70)	.53	1.10 (0.47 – 2.58)	.82

Sensitivity analysis of multivariable logistic regression in a 'full model' in which we added surgery, radiotherapy and polychemotherapy to the original model (with age, hospital and treatment intent as covariates) to gain insight in the role of treatment characteristics on outcomes.

© 2023 Baltussen JC et al. *JAMA Network Open*.

eReferences

1. Jones D, Song X, Mitnitski A, Rockwood K. Evaluation of a frailty index based on a comprehensive geriatric assessment in a population based study of elderly Canadians. *Aging Clin Exp Res.* 2005;17(6):465-471.
2. Nishijima TF, Shimokawa M, Esaki T, Morita M, Toh Y, Muss HB. A 10-Item Frailty Index Based on a Comprehensive Geriatric Assessment (FI-CGA-10) in Older Adults with Cancer: Development and Construct Validation. *Oncologist.* 2021;26(10):e1751-e1760.

© 2023 Baltussen JC et al. *JAMA Network Open*.
