# Supplementary material for: Chemotherapy-Related Toxic Effects and Quality of Life and Physical Functioning in Older Patients
Source: JAMA Netw Open. 2023 Oct 23;6(10):e2339116. doi: 10.1001/jamanetworkopen.2023.39116 (PMC10594146; doi:10.1001/jamanetworkopen.2023.39116)
Supplement: Supplement 2. — Data Sharing Statement [file jamanetwopen-e2339116-s002.pdf]

Data Sharing Statement

Baltussen. Chemotherapy-Related Toxic Effects and Quality of Life and Physical Functioning in Older Patients. *JAMA Netw Open*. Published October 23, 2023.
doi:10.1001/jamanetworkopen.2023.39116

Data
Data available: Yes
Data types: Deidentified participant data
How to access data: The dataset generated during and/or analysed during the current study are not publicly available due to participant privacy but are available from the corresponding author (J.E.A. Portielje) on reasonable request.
When available: With publication

Supporting Documents
Document types: None

Additional Information
Who can access the data: The dataset generated during and/or analysed during the current study are not publicly available due to participant privacy but are available from the corresponding author (J.E.A. Portielje) on reasonable request.
Types of analyses: any purpose
Mechanisms of data availability: After approval of a proposal by the corresponding author, with investigator support and with a signed data access agreement
